# Multi-Omics Dissection of the Shared Genetic Architecture Between Sleep Traits and Epilepsy

**DOI:** 10.3390/biology15110892

**Published:** 2026-06-05

**Authors:** Tao Wang, Jun Li, Dinghao Chen, Yunbao Liu, Canteng Fang, Xinyue Wang, Zhenjue Song, Minyu Guo, Yubo Wang, Nenad Naumovski, Xing Zheng

**Affiliations:** 1 Key Laboratory of Laboratory Medicine, Ministry of Education, Institute of Genomic Medicine, Wenzhou Medical University, Wenzhou 325035, China; 2 Zhejiang Provincial Key Laboratory of Medical Genetics, Key Laboratory of Laboratory Medicine, Ministry of Education, School of Laboratory Medicine and Life Sciences, Wenzhou Medical University, Wenzhou 325035, China; 3 School of Biomedical Engineering, Wenzhou Medical University, Wenzhou 325027, China; 4 Department of Neurology, Beijing Tiantan Hospital, Capital Medical University, Beijing 100070, China; 5 Food, Chemical and Biotechnology Cluster, Singapore Institute of Technology, Singapore 828608, Singapore; 6 School of Medicine and Psychology, Australian National University, Australian Capital Territory, Canberra 2605, Australia; 7 Department of Nutrition and Food Hygiene, School of Public Health, Wenzhou Medical University, Wenzhou 325035, China; 8 Cixi Biomedical Research Institute, Wenzhou Medical University, Ningbo 315302, China

**Keywords:** sleep behaviors, epilepsy, genetic architecture, pleiotropy effect, causal relationship

## Abstract

Epilepsy is a heritable neurological disorder that is frequently comorbid with sleeping difficulties, including short/long sleep duration and insomnia. Although epidemiological studies have consistently reported the comorbidity between sleep disturbances and epilepsy, the shared genetic architecture and molecular mechanisms underlying this relationship remain poorly characterized, hindering therapeutic development. In this study, we integrated large-scale genome-wide association study (GWAS) summary statistics of European ancestry to dissect the genetic and molecular links between sleep traits and epilepsy. Using LDSC and GWAS-pw, we identified modest but statistically significant (Bonferroni-corrected) global and local genetic correlations between sleep behaviors and epilepsy. Subsequent CPASSOC cross-trait meta-analysis and transcriptome-wide association studies (TWAS) pinpointed specific pleiotropic loci and shared candidate genes, including *SPAG7*, *VRK2*, and *LINC00925*, which are functionally associated with neuroimmune signaling. While preliminary Phenome-Wide Association Study (PheWAS) profiling of these candidate targets did not identify major adverse associations in current databases, we emphasize that rigorous in vitro and in vivo experimental validations are required before considering them for therapeutic strategies. Finally, pleiotropy-robust bidirectional Mendelian Randomization (MR) analyses suggested unidirectional causal liability from epilepsy to short sleep duration. Although the estimated causal effect size was minimal, it reflects lifelong polygenic architecture rather than acute clinical magnitude. In conclusion, our multi-omics approach unveils the shared genetic architecture of the sleep-epilepsy axis and highlights potential biomarkers for future functional investigation.

## Simple Summary

Epilepsy and sleep disturbances frequently co-occur, yet the biological mechanisms underlying their comorbidity remain poorly understood. Analyzing large-scale human genetic data, we discovered a modest but significant genetic overlap between epilepsy, short sleep, and insomnia. By integrating multi-omics data, we identified shared candidate genes, including *SPAG7*, *VRK2*, and *LINC00925*, which are active in brain tissues. Furthermore, Mendelian randomization analyses suggested a potential unidirectional causal effect of epilepsy liability on short sleep duration. These findings highlight novel genetic biomarkers and inform future therapeutic research.

## 1. Introduction

Epilepsy is a prevalent chronic neurological disorder characterized by recurrent, unprovoked seizures, posing a substantial global health burden [1,2,3]. While traditional clinical perspectives have heavily focused on disease management and drug resistance [4,5], recent advances have increasingly shifted attention toward the underlying etiology. Epilepsy is highly heterogeneous, with up to 70–80% of cases possessing a strong genetic foundation, particularly within generalized and focal forms [6,7,8]. Despite recent progress in understanding these heritable components**,** the full genetic architecture of epilepsy remains highly complex and largely elusive [9]. This complexity suggests that epileptogenesis is driven by interconnected biological networks rather than isolated molecular pathways. Therefore, deciphering the genetic overlap between epilepsy and strongly correlated phenotypic modifiers, such as sleep disturbances, may provide important insights into shared pleiotropic mechanisms.

The advent of high-density microarrays and next-generation sequencing has substantially advanced the genetic understanding of the disorder [10]. Early genome-wide association studies (GWAS) identified key susceptibility risk alleles, such as those within the *SCN1A* locus [11]. Subsequent large-scale studies have uncovered multiple genome-wide significant loci and gene variants, highlighting both shared and subtype-specific genetic architectures [9,12,13]. However, focusing solely on primary epilepsy phenotypes may overlook the broader genetic interactions between epilepsy and related phenotypic traits. Notably, the clinical manifestation of epilepsy is frequently complicated by sleep disturbances, suggesting that investigating the shared genetic components between these tightly linked traits could provide a novel approach to deciphering this genetic complexity.

Sleep is essential for maintaining physiological and neurological homeostasis, and chronic sleep disruption is inextricably linked to various adverse health outcomes [14,15]. Sleep quality is typically evaluated using core characteristics, such as sleep duration and insomnia. Individuals sleeping less than 7 h are classified as short sleepers, while those exceeding 9 h are considered long sleepers [16]. Insomnia, characterized by difficulty falling or staying asleep, demonstrates moderate genetic heritability, with single-nucleotide polymorphism (SNP)-based estimates indicating approximately 7% [17].

Findings from multiple large-scale GWAS have firmly established sleep traits as highly polygenic. These extensive meta-analyses have successfully identified numerous genome-wide significant loci regulating both short and long sleep duration, as well as insomnia, providing a robust foundation for cross-trait genetic investigations [18,19,20,21,22].

Clinically, there is substantial evidence linking sleep disturbances to epilepsy. Rather than representing independent comorbidities, sleep disturbances may modulate seizure susceptibility and disease severity. Insomnia is highly prevalent among individuals living with epilepsy, and up to 20% of seizures occur during sleep, which further exacerbates sleep fragmentation and seizure frequency [23,24]. Conversely, epilepsy disrupts circadian rhythms and synaptic protein oscillations, thereby potentially increasing neuronal excitability during sleep [25]. While observational data strongly supports this bidirectional relationship, genetic evidence remains inconclusive. Previous Mendelian randomization (MR) studies investigating causal links have yielded mixed results, with some reporting no significant associations and others suggesting partial causal relationships [26,27]. Consequently, the precise genetic and molecular connections underlying the sleep-epilepsy axis remain to be fully elucidated.

**Figure 1:**
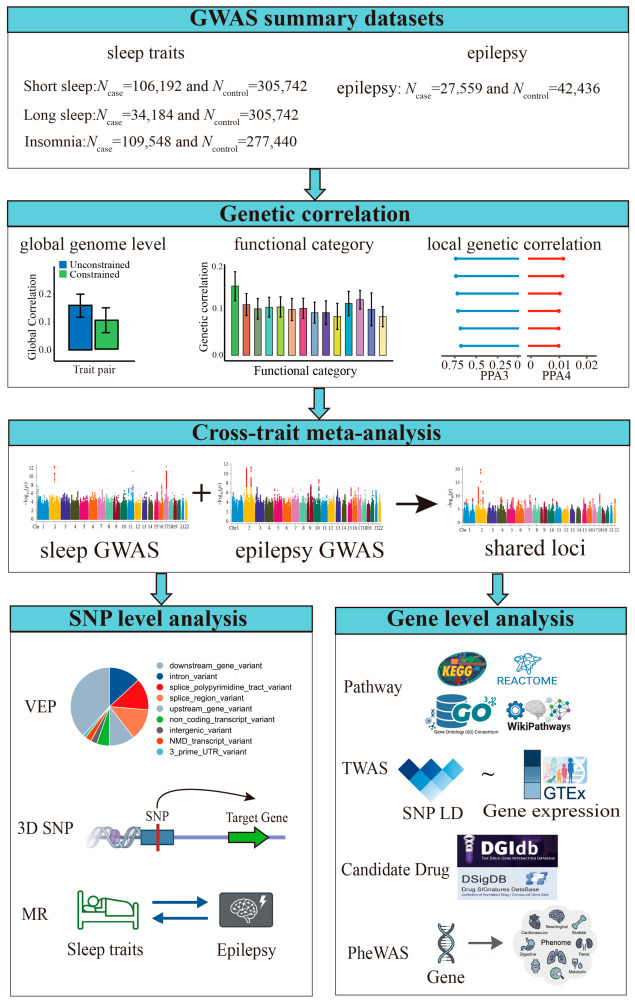
Analytical framework of the study. This workflow begins with the acquisition of GWAS summary statistics for short sleep, long sleep, insomnia, and epilepsy. Genetic correlations were then evaluated between each sleep trait and epilepsy at the genome-wide level, within functional categories, and across local genomic segments. A cross-trait meta-analysis was subsequently performed to identify shared risk loci. Finally, SNP-level annotation and gene-based analyses were conducted to uncover biological pathways underlying the genetic overlap between sleep regulation and epilepsy.

The aim of this study was to explore the shared genetic basis of sleep duration, insomnia, and epilepsy, and to identify shared genetic pathways that may inform future therapeutic strategies. To achieve this, we employed a multi-omics approach integrating GWAS, transcriptome-wide association studies (TWAS), and MR to systematically dissect the genetic overlap between sleep regulation and epilepsy. The overall analytical framework is depicted in Figure 1.

## 2. Materials and Methods

### 2.1. Sleep Duration GWAS Data

Sleep duration phenotypes were characterized using the UK Biobank participants of European ancestry. Total sleep duration, including naps (based on a 24 h cycle), was recorded in whole-hour units. The extreme values (less than 3 h or more than 18 h) were excluded from the analysis. Sleep duration was examined both as a continuous variable and in categorical form: short (<7 h), normal (7–8 h), and long (≥9 h). The GWAS were conducted separately for short sleep (106,192 cases) and long sleep (34,184 cases) individuals, using 305,742 normal-sleep individuals as controls [21]. These analyses identified 27 and 8 independent significant loci, respectively. Summary-level data are publicly accessible via the Sleep Disorder Knowledge Portal (https://sleep.hugeamp.org, accessed on 21 May 2025). Basic characteristics and SNP-based heritability estimates for both the short and long sleep duration GWAS datasets are provided in Supplementary Table S1. While these sample sizes are robust, the strict restriction to European ancestry cohorts limits the generalizability of our findings across diverse global populations, a limitation that must be considered when interpreting the results.

### 2.2. Insomnia GWAS Data

People living with insomnia and healthy controls were identified through a questionnaire that asked participants a specific question about their sleep: *‘Do you have trouble falling asleep at night or do you wake up in the middle of the night?’*. Participants selected one of four response options: *‘never/rarely’*, *‘sometimes’*, *‘usually’*, or *‘prefer not to answer’*. After excluding those who chose ‘prefer not to answer’, in total, 386,988 individuals remained included in the study. Only respondents who chose *’usually’* were classified as living with insomnia (*N* = 109,548), while all others were pooled and served as controls (*N* = 277,440). We acknowledge that relying on a single questionnaire item represents a crude phenotypic definition, which inherently carries a potential risk of misclassification bias compared to polysomnography-validated clinical diagnoses. The GWAS was then conducted on insomnia and control groups with PLINK 1.9, a whole-genome association analysis toolset, using logistic regression adjusted for age, sex, genotyping array, and the first ten genetic principal components [22]. In this study, 14 significant risk loci were identified [22], and relevant GWAS datasets are available for download at https://ctg.cncr.nl/software/summary_statistics/ (accessed on 21 May 2025) [22].

### 2.3. Epilepsy GWAS Data

According to the standard of diagnosis, epilepsy can be divided into genetic generalized epilepsy, focal epilepsy, and unclassified epilepsy [6,7]. In this study, all epilepsy GWAS summary statistics were obtained from the European ancestry based on 27,559 cases and 42,436 controls [13]. The epilepsy meta-analysis, including 4.9 million variants, identified four independent risk loci [13]. The corresponding GWAS datasets can be downloaded from https://www.ebi.ac.uk/gwas/ (accession IDs: GCST90271611, accessed on 21 May 2025). GWAS dataset description and SNP-based heritability estimates are listed in Supplementary Table S1.

### 2.4. Data Preprocessing and Quality Control

The preprocessing of the downloaded GWAS datasets was applied according to the following process. Firstly, the reference genome of all GWAS datasets was unified to GRCh37/hg19. Although GRCh38 is the latest genome assembly, hg19 remains widely used in several established analytical pipelines and annotation resources, including the clumping SNP after CPASSOC, specific pre-computed expression weight panels for TWAS, and so on. Secondly, the non-standard SNPs without the beginning of ‘rs’ were filtered. Thirdly, only the SNPs with single-nucleotide variants and filtered indels were kept. Finally, SNPs located on sex chromosomes or with a Minor Allele Frequency (MAF) < 0.01 were excluded.

### 2.5. Genome-Wide Genetic Correlation

The genome-wide genetic correlation was quantified between complex traits by applying linkage disequilibrium score regression (LDSC) to publicly available GWAS summary statistics, thereby avoiding the need for individual-level genotype data [28]. The LDSC exploited the non-random co-inheritance of alleles captured by linkage disequilibrium and assumed that single-trait GWAS χ^2^ statistics are inflated for SNPs located in regions of strong LD. Cross-trait LDSC extended this framework by replacing the χ^2^ value with the product of the signed z-scores obtained from two independent GWAS, yielding an estimate of genetic covariance [28]. While LDSC inherently relies on specific polygenicity assumptions, we utilized unconstrained intercept models to account for potential biases arising from unknown sample overlaps between the datasets**.** Each summary dataset was first filtered to retain well-imputed HapMap 3 variants and then aligned to the European ancestry panel of the 1000 Genomes Phase 3 reference before global correlation calculation [29].

To ensure data reliability, we applied the default filtering parameters of LDSC, specifically removing SNPs with an imputation quality (INFO) score ≤ 0.9, to calculate genetic correlation. Futhermore, to guard against multiple-testing inflation, a strict Bonferroni correction was applied so that associations between sleep duration, insomnia, and epilepsy were deemed significant only when *p* < (0.05/3 = 0.0167), properly accounting for the three primary sleep phenotypic categories tested against epilepsy.

### 2.6. Local Genetic Overlap

Although LDSC remains the tool of choice for estimating the genome-wide genetic correlation between two complex traits, its resolution does not indicate whether covariance originates uniformly across the genome or is driven by particular chromosomal segments [30]. To determine the magnitude of genetic overlap within defined loci, GWAS-pw was used to quantify the local genetic correlation [31,32].

The GWAS-pw, based on a Bayesian statistical framework, examined 1703 linkage disequilibrium-independent genomic regions, assessing whether pairs of traits share co-localized genetic signals [32]. Within each segment, posterior probabilities for four mutually exclusive scenarios were provided: PPA1, association exclusive to the first trait; PPA2, association exclusive to the second trait; PPA3, a single variant that simultaneously influences both traits; and PPA4, two distinct causal variants, one for each trait [33]. The operationality interpreted PPA3 > 0.5 as decisive support for shared genetic control at the locus, whereas PPA4 > 0.5 was taken to indicate the presence of two independent, trait-specific associations within the same genomic window [34]. Because the PPA represents a Bayesian posterior probability that directly quantifies the likelihood of a specific structural model given the data, rather than a frequentist *p*-value, standard frequentist multiple-testing adjustments (such as Bonferroni or False Discovery Rate) are inherently inapplicable. A PPA threshold > 0.5 is a mathematically established benchmark in the literature providing robust evidence for colocalization without requiring further multiplicity penalization across the 1703 regions [32,34].

### 2.7. Partitioned LDSC

The continuum of the genome was deconstructed into fourteen discrete functional compartments, and partition-based LDSC was used to quantify the proportion of genetic covariance that falls within each. The catalogue spans enhancers, super-enhancers, the transcribed territory, transcription-factor binding sites (TFBS), introns, DNase-I digital genomic footprinting (DGF) segments, DNase-I hypersensitive sites (DHSs), fetal-stage DHSs, the activating histone marks H3K9ac, H3K4me1, H3K4me3, and H3K27ac, together with canonically repressed regions [35]. For every compartment, annotation-specific LD scores were generated. These bespoke metrics were then inserted into the regression framework to produce heritability and correlation estimates that are conditional on the functional label. To insulate the inference from multiple-testing inflation, strict Bonferroni correction (*p* value < 0.05/14) was applied to rigorously account for the 14 independent functional categories evaluated, and the explicitly adjusted *p*-values are reported in the Supplementary Tables.

### 2.8. Cross-Trait Association Analysis

To identify variants jointly influencing multiple phenotypes, a cross-trait meta-analysis with Cross-Phenotype Association (CPASSOC) was performed [36]. This summary-statistic approach evaluates whether an SNP’s combined effect across traits exceeds its marginal effect on any single trait, thereby detecting loci that exert pleiotropic effects.

The CPASSOC uses two algorithms, Shom and Shet [36]. Shom treats the SNP effect as fixed and identical across traits, making it appropriate only when allelic effects are both concordant and of comparable magnitude. In contrast, Shet allows the per-allele effect to vary in size or direction among traits by fitting a random-effects model that up-weights informative phenotypes and down-weights those with negligible or heterogeneous effects [36,37]. Given the distinct physiological architectures of sleep traits and epilepsy, significant genetic heterogeneity and directionally discordant allelic effects are highly anticipated. The Shet statistic is mathematically optimized to maintain statistical power under such heterogeneous conditions, making it superior to the Shom model for our specific multi-trait analysis; therefore, Shet was adopted as the primary test.

After cross-trait meta-analysis, a number of genetic variants shared by two traits were identified. To further ascertain the independent pleiotropic SNPs, PLINK 1.9 was used to clump the SNPs with a primary significance threshold of *p* < 5 × 10^−8^ (-clump-p1 5e-8), a secondary significance threshold of *p* < 1 × 10^−5^ (-clump-p2 1e-5), an LD cutoff of *r*^2^ = 0.001 (-clump-r2 0.001), and a clumping window of 500 kb (-clump-kb 500). We acknowledge that while these highly stringent clumping parameters are necessary to minimize false positives, this conservative approach presents a trade-off that may inadvertently omit weaker, yet biologically relevant, pleiotropic signals. The genetic variant with the lowest *p* value within each LD block was selected as the index SNP. The SNPs that simultaneously meet the criteria of *p* value less than 5 × 10^−8^ after meta-analysis and a *p* value less than 0.05 for a single trait are considered significant pleiotropic loci [38]. In order to further obtain the function annotation of significant pleiotropic loci, the Ensembl Variant Effect Predictor (VEP) tool (asia.ensembl.org)and the 3DSNP database for linking noncoding SNPs to their three-dimensional interacting genes (https://3dsnp.omic.tech/, accessed on 2 July 2025) were applied to obtain the nearest gene based on physical proximity [39,40]. In addition, 3DSNP was used to obtain the regulated target gene based on chromatin three-dimensional interactions [40].

### 2.9. Pathway Enrichment Analysis

To clarify the biological roles of pleiotropic SNPs, their annotated genes, including nearest genes, three-dimensional interacting genes, and genes residing within the clumping interval, were extracted, and the combined list was submitted to the web platform Metascape (accessed on 15 July 2025) for pathway enrichment, retaining all default settings [41].

### 2.10. Transcriptome-Wide Association Study

Cross-trait meta-analysis pinpoints SNPs that influence both phenotypes, yet most risk variants act by modulating gene expression. To highlight the genes whose regulation is jointly implicated, a transcriptome-wide association study (TWAS) with GTEx v8 expression weights using the FUSION tool (http://gusevlab.org/projects/fusion/, accessed on 15 July 2025) was performed [33]. TWAS statistics were initially computed across whole blood and 13 brain tissues. However, to ensure biological relevance, downstream interpretation and candidate gene prioritization were heavily emphasized within epilepsy-relevant central nervous system tissues, specifically the hippocampus and frontal cortex. Gene–tissue pairs surviving False Discovery Rate (FDR) correction within their respective tissues were retained. Shared genes were defined as those exhibiting significant expression associations with both sleep traits and epilepsy in at least one common tissue.

### 2.11. Genetic Drug Target Analysis

To prioritize potential therapeutic drugs, we queried the Drug–Gene Interaction Database (DGIdb) [42] and DSigDB (accessed on 20 July 2025) [43] for FDA-approved compounds whose targets overlap the shared genes, yielding a pharmacological background that can inform treatment strategies for both short/long sleep duration, insomnia, and epilepsy [42,43]. Identified non-coding loci lacking established pharmacological interactions were strictly classified as mechanistic candidates rather than direct therapeutic targets.

### 2.12. Phenome-Wide Association Study

A phenome-wide association study (PheWAS) systematically relates a given variant or gene to the full spectrum of phenotypic outcomes recorded in large biobanks, thereby revealing pleiotropic effects. Using the AstraZeneca PheWAS portal (https://www.azphewas.com/, accessed on 26 July 2025), which is built on UK Biobank exome and phenotype data, the disease landscape linked to each drug-target gene to flag potential *on-* or *off*-target liabilities of the corresponding compounds was screened [44]. A strict phenome-wide significance threshold (Bonferroni-corrected *p* < 0.05/total number of traits tested) was applied to determine potentially significant adverse associations. Importantly, while we screened for off-target effects, we strictly operate under the premise that the absence of evidence for adverse traits in current databases does not constitute definitive evidence of clinical safety.

### 2.13. Mendelian Randomization

The MR uses genetic variants as instrumental variables to estimate whether an exposure causally influences an outcome. Valid instruments must satisfy three conditions: they are robustly associated with the exposure, independent of confounders, and affect the outcome only through the exposure [45]. A bidirectional two-sample MR was performed to examine causal effects in both directions between short/long sleep duration, insomnia, and epilepsy.

In this study, genetic variants were selected as IVs based on the following criteria: (1) SNPs are strongly associated with the exposure (*p* < 5 × 10^−8^) from GWAS; (2) keep the independent SNPs with an LD *r*^2^ < 0.001 (window size = 10 Mb) according to the 1000 Genomes phase 3 reference panel; and (3) SNPs demonstrating sufficient instrument strength, as indicated by an F-statistic exceeding 10.

A suite of complementary MR estimators was applied. The inverse-variance-weighted (IVW) estimator served as the primary analysis [46]. To rigorously validate our causal inference and ensure compliance with core MR assumptions, standard heterogeneity and pleiotropy tests were subsequently performed. Cochran’s Q test was utilized to quantify heterogeneity among the selected SNPs; in instances of significant heterogeneity, a random-effects IVW model was employed. Furthermore, the MR-Egger intercept test was applied to detect and statistically adjust for unbalanced horizontal pleiotropy, ensuring that the directional causal estimates were not biased by alternative biological pathways. The weighted-median, simple-mode, and weighted-mode estimators were additionally employed to relax instrumental validity requirements and improve robustness. Every MR test was executed with TwoSampleMR (version 0.5.7) in the R environment (version 4.3.2), ensuring full reproducibility of the workflow [47].

## 3. Results

### 3.1. Genome-Wide Heritability and Global Genetic Correlation

The SNP-based heritability estimated by univariate LDSC was 0.0495 for short sleep duration, 0.029 for long sleep duration, 0.046 for insomnia, and 0.1049 for epilepsy (Supplementary Table S1). It is important to note that these heritability estimates are relatively low, accompanied by high standard deviations in the summary statistics. This substantial variability is characteristic of extremely polygenic traits, reflecting the profound clinical and environmental heterogeneity inherent to both sleep behaviors and epilepsy. Consequently, these estimates possess limited explanatory power for deterministic single-gene effects and should be interpreted as representing a broad, highly distributed polygenic liability.

**Figure 2:**
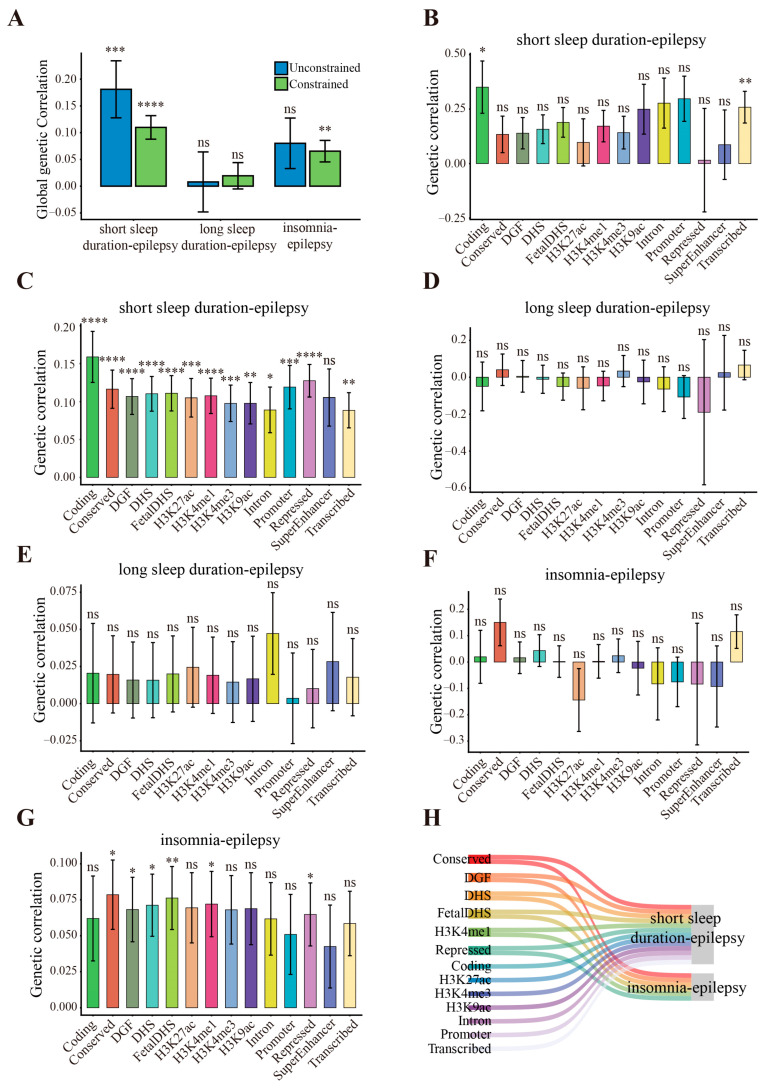
Global and local genetic correlations between sleep traits and epilepsy. (**A**) Global genetic correlations between short/long sleep duration, insomnia, and epilepsy, estimated with both unconstrained (blue color) and constrained (green color) LDSC. The x-axis represents trait pairs, and the y-axis shows the genetic correlation coefficient. (**B**–**G**) Partitioned genetic correlations of short sleep duration (**B**,**C**), long sleep duration (**D**,**E**), and insomnia (**F**,**G**) with epilepsy across functional categories, analyzed using unconstrained (**B**,**D**,**F**) and constrained (**C**,**E**,**G**) LDSC, respectively. (**H**) Sankey diagram summarizing significant partitioned genetic correlations among short sleep duration, insomnia, and epilepsy. Unconstrained = LDSC without constrained intercept; Constrained = LDSC with constrained intercept. Significance thresholds: ns = *p* > 0.05/14; * = *p* < 0.05/14; ** = *p* < 0.01/14; *** = *p* < 0.001/14; **** = *p* < 0.0001/14. In panels (**B**–**G**), distinct colors are used to visually differentiate the 14 functional categories. In panel (**H**), the colored bands denote significant partitioned genetic correlations, linking specific functional categories to their corresponding sleep–epilepsy trait pairs. Abbreviations: DGF, DNase I digital genomic footprinting; DHS, DNase I hypersensitive sites.

Global genetic correlation analysis consistently revealed a modest but significant positive correlation between short sleep duration and epilepsy across both unconstrained (*r_g_* = 0.181, 95% CI: [0.077, 0.285], *p* = 7.00 × 10^−4^) and constrained (*r_g_* = 0.1098, 95% CI: [0.067, 0.153], *p* = 5.76 × 10^−7^) LDSC models (Figure 2A, Supplementary Table S2). This robust consistency underscores a definitive shared global genetic basis between the two traits. Conversely, no significant associations were observed for long sleep duration under either model. For insomnia, while the unconstrained model showed no significant association, constrained LDSC identified a significant genetic correlation (*r_g_* = 0.0654, 95% CI: [0.026, 0.105], *p* = 1.10 × 10^−3^).

In summary, there was a significant global genetic correlation between short sleep duration and epilepsy, regardless of LDSC intercept constraints, and between insomnia and epilepsy using constrained LDSC. The discrepancy observed between unconstrained and constrained LDSC models for insomnia is likely reflected by the constrained model’s enhanced statistical power. By robustly accounting for potential sample overlap between the massive UK Biobank and the epilepsy consortium cohorts, the constrained model captures true shared biological variance that the unconstrained model was underpowered to detect. Biologically, the stronger genetic correlation observed for short sleep duration compared to long sleep duration suggests distinct underlying architectures. Short sleep is frequently associated with heightened systemic arousal and network excitability, which may contribute to seizure generation. In contrast, long sleep may be more closely tied to secondary systemic factors, such as inflammation or metabolic dysregulation.

### 3.2. Partitioned Genetic Correlation by Functional Category

To explore the genetic correlations between short/long sleep duration, insomnia, and epilepsy across different functional regions of the genome, partitioned LDSC genetic correlations for 14 functional categories by partitioning LDSC were calculated, with significance strictly defined by a Bonferroni-adjusted threshold (*p* < 0.05/14). The explicitly adjusted *p*-values are detailed in Supplementary Tables S3–S5.

The correlations between short sleep duration and epilepsy exhibited significant associations in coding and transcribed regions using unconstrained LDSC (Figure 2B, Supplementary Table S3). With constrained LDSC, significant correlations were observed in coding, conserved, DGF, DHS, fetal DHS, H3K27ac, H3K4me1, H3K4me3, H3K9ac, intron, promoter, repressed, and transcribed regions (Figure 2C, Supplementary Table S3). Notably, the strongest correlation was found in the coding region (*r*_g_ = 0.3508, 95% CI: [0.116, 0.586]), which is critical for gene expression and protein synthesis [48]. Rather than a generalized genomic overlap, this pronounced correlation within coding/transcribed regions suggests that the shared genetic liability directly impacts protein sequence and function, likely altering specific synaptic signaling or neurodevelopmental pathways that simultaneously govern sleep-wake cycles and seizure thresholds.

There were no significant correlations found for the long sleep duration and epilepsy using either unconstrained or constrained LDSC (Figure 2D,E, Supplementary Table S4). Regarding insomnia and epilepsy, unconstrained LDSC did not reveal significant correlations (Figure 2F, Supplementary Table S5), but constrained LDSC identified significant correlations in conserved, DGF, DHS, fetal DHS, H3K4me1, and repressed regions (Figure 2G, Supplementary Table S5). The strongest correlation was found in the conserved region (*r_g_* = 0.0785, 95% CI: [0.031, 0.126]), which is evolutionarily preserved across species [49].

These findings are also summarized in Figure 2H, highlighting the significant partitioned genetic correlations between short sleep duration, insomnia, and epilepsy.

### 3.3. Local Genetic Correlation

Given the significant genetic correlations observed at the global and functional levels, specific genomic regions that contributed to local genetic overlap were analyzed using GWAS-pw.

**Figure 3:**
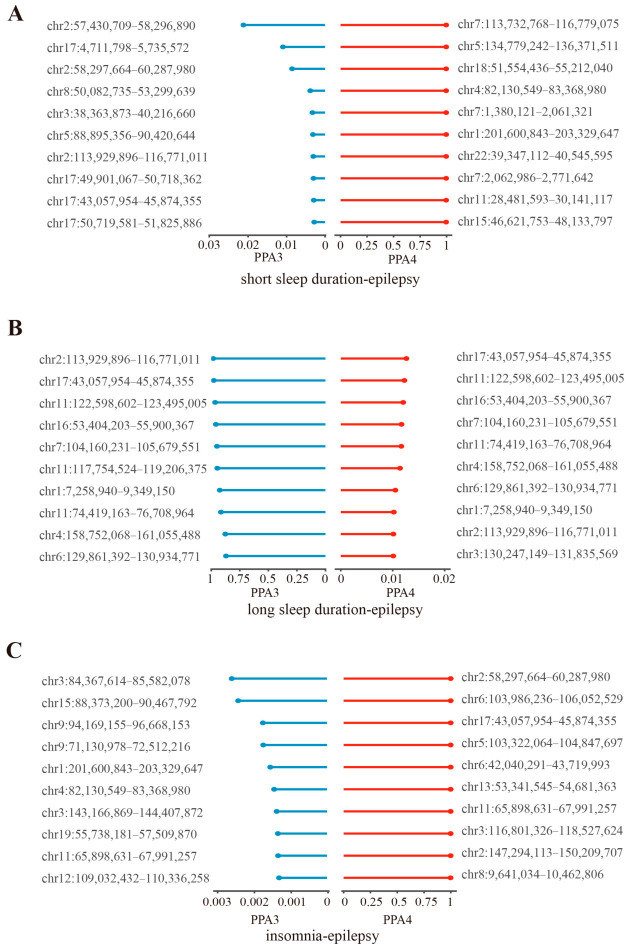
Local genetic correlations between sleep traits and epilepsy. (**A**–**C**) Local genetic overlap mapping across 1703 LD-independent regions identifies specific chromosomal segments driving the association between (**A**) short sleep, (**B**) long sleep, and (**C**) insomnia with epilepsy, highlighting distinct regional contributions to the shared genetic architecture. Blue lines indicate the posterior probability of a single shared causal variant (PPA3) influencing both traits, whereas red lines represent the posterior probability of two distinct, trait-specific causal variants (PPA4).

Partitioning the genome into 1703 independent regions revealed no shared loci (PPA3 > 0.5) between short sleep duration and epilepsy (Figure 3A, Supplementary Table S6). However, over ten regions showed distinct, independent associations for these traits (PPA4 > 0.5).

For the correlations between the long sleep duration and epilepsy, more than ten genomic regions exhibited significant shared associations (PPA3 > 0.5), including chr2:113,929,896–116,771,011, chr17:43,057,954–45,874,355, chr11:122,598,602–123,495,005, chr16:53,404,203–55,900,367, chr7:104,160,231–105,679,551, chr11:117,754,524–119,206,375, chr1:7,258,940–9,349,150, chr11:74,419,163–76,708,964, chr4:158,752,068–161,055,488, chr6:129,861,392–130,934,771, and several others (Figure 3B, Supplementary Table S7).

No significant shared loci (PPA3 > 0.5) were detected between insomnia and epilepsy (Figure 3C, Supplementary Table S8), though more than ten regions showed distinct associations (PPA4 > 0.5).

### 3.4. Pleiotropic Loci Identified by Cross-Trait Meta-Analysis

**Figure 4:**
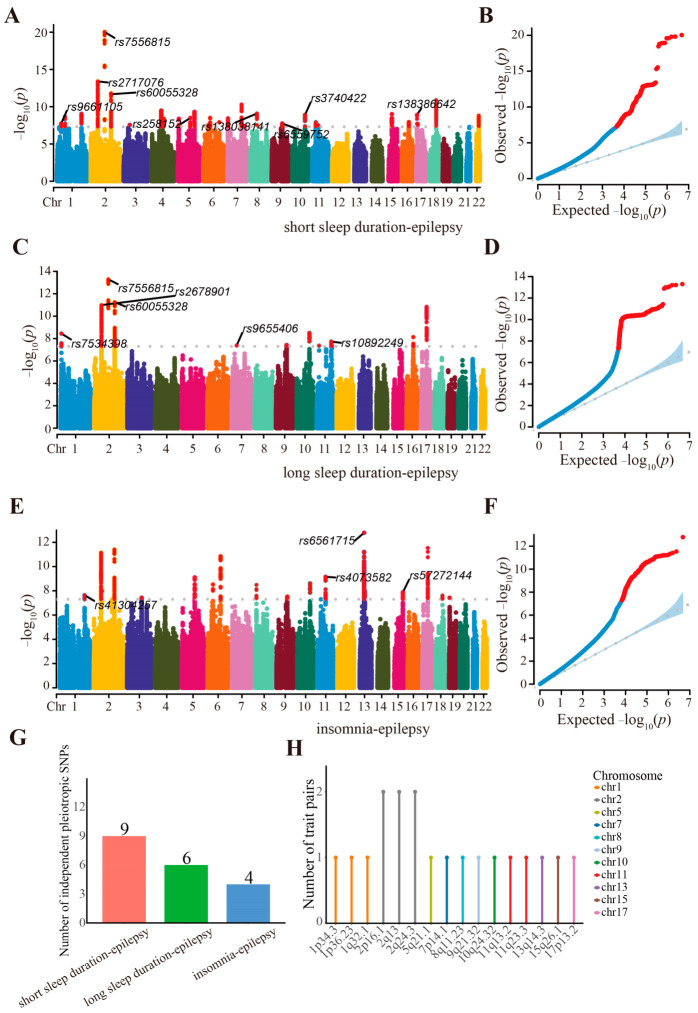
Pleiotropic loci shared between sleep behaviors and epilepsy. Manhattan and QQ plots illustrate independent pleiotropic SNPs linking short sleep (**A**,**B**), long sleep (**C**,**D**), and insomnia (**E**,**F**) with epilepsy. (**G**,**H**) Summaries of shared loci indicate the key chromosomal regions mediating these cross-trait associations. Index SNPs are labeled in black; gray dashed lines in the Manhattan plots denote the genome-wide significance threshold. In the QQ plots, red points highlight SNPs that surpass the genome-wide significance threshold, while blue points denote non-significant variants. chr: chromosome.

A genome-wide cross-trait meta-analysis was performed to identify pleiotropic loci shared between short/long sleep duration, insomnia, and epilepsy using CPASSOC (*P*_CPASSOC_ < 5 × 10^−8^ and *P*_single-trait_ < 0.05). After pruning, nine independent pleiotropic loci associated with short sleep duration and epilepsy (Figure 4A,B, Supplementary Table S9), six loci for long sleep duration and epilepsy (Figure 4C,D, Supplementary Table S10), and four loci for insomnia and epilepsy were identified (Figure 4E,F, Supplementary Table S11).

Importantly, several of these identified loci strongly overlap with established biological pathways regulating neuronal excitability. For short sleep duration and epilepsy, the first locus (index SNP: rs7556815, *P*_CPASSOC_ = 9.12 × 10^−21^) was near *LOC101927400*, a gene linked to sleep duration [50]. The second locus (rs2717076, *P*_CPASSOC_ = 3.94 × 10^−14^) and third locus (rs60055328, *P*_CPASSOC_ = 1.60 × 10^−12^) were near *SCN1A* and *TTC21B*. SCN1A is a highly characterized epilepsy susceptibility gene governing voltage-gated sodium channels, thereby reinforcing the biological validity of our cross-trait findings [51]. The *TTC21B* is located on the axoneme of cilia and may play a role in the retrograde axoneme transport process of cilia. The mutation of this gene is associated with various ciliopathies, renal tubular atrophy syndrome, and restrictive thoracic myopathy [52,53]. The fourth locus (rs138038141, *P*_CPASSOC_ = 8.09 × 10^−10^) was near *PXDNL*, a gene involved in heme binding and peroxidase activity [54]. The fifth locus (rs138386642, *P*_CPASSOC_ = 1.20 × 10^−9^) was located near *KIF1C*, which encodes a microtubule-dependent motor protein essential for organelle transport and chromosome movement [55]. The sixth locus (rs3740422, *P*_CPASSOC_ = 1.31 × 10^−9^) was close to *MGEA5*, a glycosidase that removes O-GlcNAc modifications [56]. Additional shared loci included rs258152 (near *PAM*), rs6559752 (*C9orf64*), and rs9661105 (*LOC105378639*).

For long sleep duration and epilepsy, loci rs7556815 (*P*_CPASSOC_ = 5.08 × 10^−14^) near *LOC101927400*, and rs60055328 (*P*_CPASSOC_ = 5.63 × 10^−12^) near *SCN1A* and *AC010127.3*, an antisense RNA for *SCN9A*, were identified [57]. The third locus (index SNP: rs2678901, *P*_CPASSOC_ = 1.02 × 10^−11^) was near *VRK2*, which encodes a serine-threonine kinase that modulates MAPK signaling and synaptic pruning. These mechanisms are intimately linked to neurodevelopmental structural integrity, providing a clear biological rationale for their reported associations with neurological disorders such as epilepsy and schizophrenia [58]. The other loci, including rs7534398 (near *CAMTA1*) and rs10892249 (near *PHLDB1* and *AP002954.3*), were also identified.

For insomnia and epilepsy, the loci rs6561715 (*P*_CPASSOC_ = 1.64 × 10^−13^) and rs4073582 (*P*_CPASSOC_ = 6.92 × 10^−10^) were identified near the *CNIH2*, *YIF1A*, and *RP11-867G23.3*. *CNIH2* acts as an auxiliary subunit of AMPA receptors, fundamentally participating in excitatory neurotransmission and synaptic plasticity [59]. The *YIF1A*, as a transmembrane protein, is involved in the maintenance of the Golgi apparatus structure [60] while *RP11-867G23.3*, an lncRNA, is associated with cancer development [61]. Two additional pleiotropic loci, including rs57272144 (*P*_CPASSOC_ = 1.33 × 10^−8^, near *LINC00925*, a long non-coding RNA associated with cellular stress pathways and ferroptosis [62], which may influence neuronal survival during epileptic seizures) and rs41304257 (*P*_CPASSOC_ = 2.28 × 10^−8^, near *IPO9*), were also identified.

In summary, 16 pleiotropic regions are shared between short/long sleep duration, insomnia, and epilepsy (Figure 4G,H). Notably, regions 2p16.1, 2q13, and 2q24.3 were associated with two trait pairs, while other regions were trait-specific.

### 3.5. Pathway Enrichment Results

**Figure 5:**
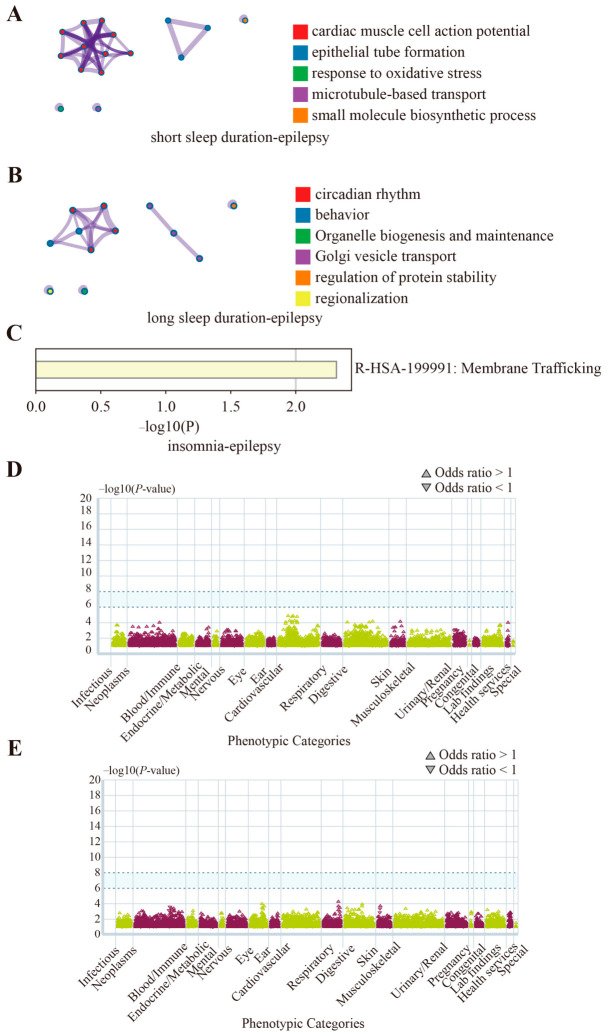
Pathway enrichment and phenome-wide associations of shared candidate genes. (**A**–**C**) Metascape pathway enrichment highlights specific biological processes, such as microtubule-based transport, circadian rhythm and membrane trafficking, underlying the genetic overlap. The nodes represent enriched terms and the edges represent similarity between terms. (**D**,**E**) PheWAS results for *SPAG7* and *VRK2* demonstrate a lack of major pleiotropic liabilities across broad phenotypic categories. The x-axis represents phenotypic categories, and the y-axis shows −log10 *p* values from the PheWAS. The upper and lower dashed lines indicate genome-wide significance (−log10 *p* = 8) and suggestive (−log10 *p* = 6) thresholds, respectively.

Using annotated genes for pleiotropic loci for short sleep duration and epilepsy, five significantly enriched pathways were identified, further contextualizing the genetic overlap. These included cardiac muscle cell action potential, epithelial tube formation, response to oxidative stress, microtubule-based transport, and small molecule biosynthetic process (Figure 5A, Supplementary Table S12). As for the long sleep duration and epilepsy trait pair, we found six enriched pathways, including circadian rhythm, behavior, organelle biogenesis and maintenance, Golgi vesicle transport, regulation of protein stability, and regionalization (Figure 5B, Supplementary Table S13). There was one significantly enriched pathway for insomnia and epilepsy, including R-HSA-199991: Membrane Trafficking (Figure 5C, Supplementary Table S14).

### 3.6. Transcriptome-Wide Association Studies

The TWAS method was used to evaluate the gene-level shared effects on the sleep behaviors and epilepsy trait pairs.

For short sleep duration and epilepsy, the corresponding genes associated with short sleep duration and epilepsy were identified (FDR-corrected *p* < 0.05), respectively. After merging the above genes, the two pleiotropic genes (*SPAG7* and *VRK2*) were identified in brain tissue. *SPAG7* was significantly identified across the cerebellar hemisphere, cerebellum, frontal cortex BA9, and hippocampus. Functionally, SPAG7 is heavily implicated in neuroimmune modulation and inflammatory responses [63]. Its robust expression in key epileptogenic zones like the hippocampus suggests a potential role in mediating neuroinflammation-induced hyperexcitability, thereby linking sleep dysregulation to altered seizure thresholds. In addition, the gene *VRK2* shared by short sleep duration and epilepsy in the brain Substantia nigra tissue was also identified (Supplementary Table S15). The above two shared genes were located at independent pleiotropic loci identified by CPASSOC.

For insomnia and epilepsy, a non-coding gene, *LINC00925*, was identified in whole blood (Supplementary Table S15). This gene was also located at independent pleiotropic loci identified by CPASSOC; however, there was no shared gene for long sleep duration and epilepsy.

### 3.7. Genetic Drug Target Results

Based on the identified shared genes, we prioritized potential candidate compounds for future investigation. *SPAG7* and *VRK2* were selected as the potential target genes for the short sleep duration and epilepsy trait pair, while *LINC00925* was selected as the target gene for insomnia and epilepsy. This was followed by the use of DGIdb and SigDB to obtain FDA-approved medication for *SPAG7*, *VRK2*, and *LINC00925*. Although there was no specific medication provided by the DSigDB, there were two medications (VORINOSTAT and VALPROIC ACID) for the target gene *SPAG7* and four medications (DASATINIB, BOSUTINIB, CHLORZOXAZONE, VORINOSTAT) for the target gene *VRK2*. Furthermore, as *LINC00925* is an lncRNA, searches within the DGIdb and DSigDB revealed no candidate medications (Supplementary Table S16). Consequently, LINC00925 is more accurately characterized as a genetic biomarker or mechanistic candidate rather than a viable therapeutic target.

### 3.8. Phenome-Wide Association Studies

The potential side effect of selected medications was assessed using the PheWAS. According to the AstraZeneca PheWAS portal and based on sequencing and phenotype data from the UK Biobank, the target genes (*SPAG7* and *VRK2*) were not significantly associated with infectious, neoplasms, blood/immune, endocrine/metabolic, mental, nervous, eye, ear, cardiovascular, respiratory, digestive, skin, musculoskeletal, urinary/renal, pregnancy, congenital, laboratory findings, health services, and special phenotypic categories (Figure 5D,E). While these findings suggest a lack of major pleiotropic liabilities within the queried phenotypes, we strictly emphasize that the absence of statistical evidence in current genetic databases does not constitute definitive evidence of clinical safety, and these candidates necessitate rigorous downstream pharmacological evaluation.

### 3.9. Mendelian Randomization Analysis of Sleep Behaviors and Epilepsy

**Figure 6:**
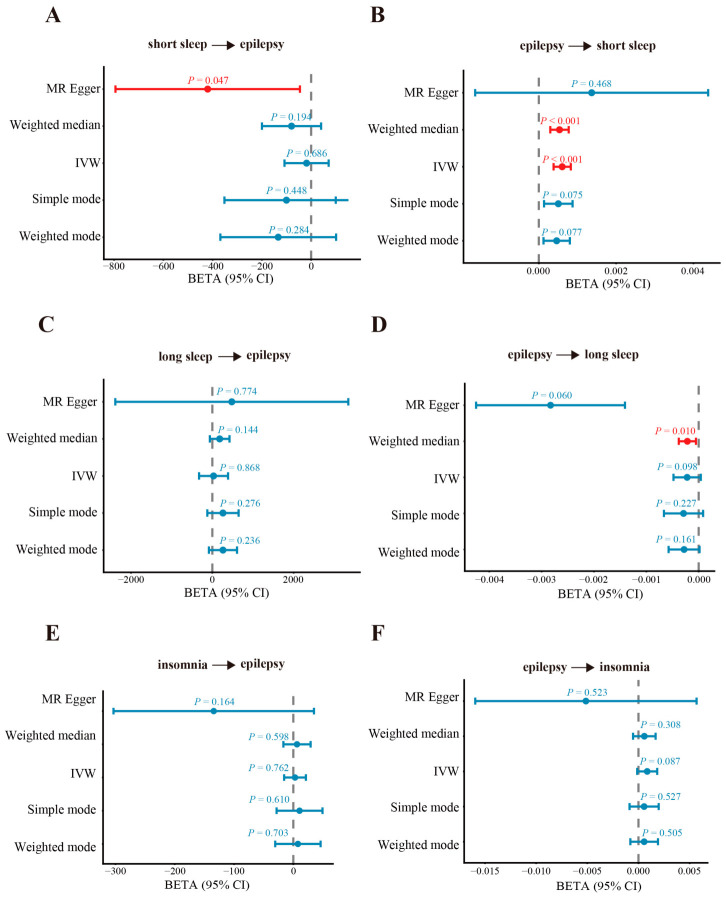
Bidirectional MR analyses. MR estimates assess causal relationships between (**A**,**B**) short sleep, (**C**,**D**) long sleep, and (**E**,**F**) insomnia with epilepsy. Significant causal liability was uniquely observed from epilepsy to short sleep duration, suggesting a unidirectional polygenic influence. The significant MR results (*p* < 0.05) are indicated in red.

A bidirectional two-sample MR was conducted to investigate potential causality between sleep behaviors and epilepsy traits. The inverse-variance weighted (IVW) was utilized as the main method to explore the potential causality. When referring to short sleep duration and epilepsy, 15 independent genetic variants associated with short sleep duration were used as IVs with *F*-statistics > 10. According to the IVW, the short sleep duration is not causally associated with epilepsy (Figure 6A). However, epilepsy can increase the risk of short sleep duration (BETA = 0.0006, *p* = 1.1 × 10^−7^) (Figure 6B). In addition, the weighted median results (BETA = 0.00054, *p* = 1.1 × 10^−5^) indicated epilepsy was significantly associated with short sleep duration (Figure 6B). While statistically robust, it is crucial to clarify that this extremely small effect size (BETA = 0.0006) is biologically minimal in an acute clinical context. It reflects a subtle, lifelong polygenic liability rather than a dramatic clinical shift. As for the long sleep duration and epilepsy, long sleep duration is not causally associated with epilepsy (IVW: BETA = 30.327, *p* = 0.868) (Figure 6C), and vice versa (IVW: BETA = −0.0002, *p* = 0.098) (Figure 6D).

In addition to short and long sleep durations, the association between insomnia and epilepsy was further investigated. The 9 independent SNPs with F statistics > 10 were used as IVs to explore the effect of insomnia on epilepsy trait pairs. According to IVW results, insomnia does not increase the risk of epilepsy (IVW: BETA = 2.838, *p* = 0.762) (Figure 6E), and vice versa (IVW: BETA = 0.0009, *p* = 0.087) (Figure 6F).

Sensitivity analyses were conducted to verify the core MR assumptions (Supplementary Table S17). MR-Egger intercept tests indicated an absence of horizontal pleiotropy across all bidirectional analyses (all *p* > 0.05), suggesting the causal estimates are not biased by alternative pathways. Additionally, Cochran’s Q tests generally showed no significant heterogeneity among the instrumental variables. For analyses involving long sleep duration where significant heterogeneity was present (Cochran’s Q *p* < 0.05), a random-effects IVW model was applied to accommodate the variance and maintain the stability of the null findings.

In summary, epilepsy was causally associated with short sleep duration, but not vice versa. Moreover, long sleep duration and insomnia were not associated with the development of epilepsy. This interpretive gap highlights distinct neurobiological architectures. While short sleep may be directly modulated by the acute hyperarousal and network excitability inherent to epileptogenesis, long sleep and insomnia likely represent secondary phenotypic consequences. These latter traits are potentially driven by other shared environmental factors or distinct psychiatric comorbidities, rather than acting as direct causal drivers within the sleep–epilepsy axis.

## 4. Discussion

Epilepsy is a common and debilitating neurological disorder that poses a substantial health and economic burden on society. Sleep traits, such as short and long sleep duration and insomnia, play a crucial role in maintaining physiological and neurological balance. While previous epidemiological and observational genomic studies have established a correlation between epilepsy and sleep disturbances, our study provides an integrative multi-omics perspective on the genetic relationship between sleep traits and epilepsy. In this study, the shared genetic basis between sleep behaviors and epilepsy was systematically explored using a range of bioinformatics approaches. Initial genetic correlations at the genome-wide level, within specific functional annotations, and across 1703 independent genomic regions were evaluated. Unlike standard analytical pipelines, our implementation of the CPASSOC algorithm explicitly accounts for genetic heterogeneity and directionally discordant effects across diverse cohorts. Findings of the genome-wide cross-trait meta-analysis revealed several pleiotropic loci shared by sleep duration, insomnia, and epilepsy, which were further functionally annotated. Additionally, TWAS, integrating GWAS and expression quantitative trait loci (eQTL) data, identified pleiotropic genes whose expression influences both traits. While candidate genes were identified, we strictly emphasize that PheWAS was performed to assess the risk of off-target effects. Finally, bidirectional MR analyses suggested a causal relationship, showing that epilepsy may lead to the development of short sleep duration.

The genetic correlation between short/long sleep duration, insomnia, and epilepsy using LDSC with both unconstrained and constrained intercepts was performed. Significant genome-wide correlations were observed for short sleep duration and epilepsy under the unconstrained model. The LDSC with constrained intercepts, which improves statistical power without sample overlap [28], further identified significant correlations between short sleep duration, insomnia, and epilepsy. We classified the genome into 14 functional categories and calculated genetic correlations within these categories. Both analyses revealed significant correlations in several categories. Additionally, we examined genetic correlations across 1703 linkage disequilibrium-independent genomic regions using GWAS-pw, identifying significant correlations (PPA3 > 0.5) for long sleep duration and epilepsy in multiple regions. Collectively, these findings provide modest but significant evidence that sleep duration and insomnia share substantial genetic overlap with epilepsy.

Our analysis revealed a shared etiology between short and long sleep duration, insomnia, and epilepsy. To explore this relationship further, we conducted a genome-wide cross-trait meta-analysis to identify pleiotropic loci associated with these trait pairs, followed by functional annotation. This identified nine, six, and four independent pleiotropic loci for short sleep duration and epilepsy, long sleep duration and epilepsy, and insomnia and epilepsy, respectively. Notably, chromosomal regions 2p16.1, 2q13, and 2q24.3 were common to two trait pairs, while regions 1p34.3, 1p36.23, 1q32.1, 5q21.1, 7p14.1, 8q11.23, 9q21.32, 10q24.32, 11q13.2, 11q23.3, 13q14.3, 15q26.1, and 17p13.2 were shared by a single trait pair. Importantly, cross-referencing our complementary analytical approaches revealed that several genomic regions identified by the CPASSOC meta-analysis showed positional overlap with the pleiotropic loci flagged by the Bayesian GWAS-pw analysis. This cross-validation between frequentist and Bayesian frameworks further reinforces the robustness of these specific shared genetic regions, suggesting that they harbor critical regulatory elements modulating synaptic plasticity and neuronal excitability.

Furthermore, the shared architecture between short/long sleep duration, insomnia, and epilepsy was further validated at the gene level by TWAS. Specifically, *SPAG7* and *VRK2* were identified as pleiotropic genes shared between short sleep duration and epilepsy, while *LINC00925* was shared between insomnia and epilepsy. Based on gene prioritization, we identified these three candidate genes (*SPAG7*, *VRK2*, and *LINC00925*), and they require comprehensive mechanistic contextualization prior to any translational considerations. The tissue-specific expression patterns of these genes elucidate their distinct functional roles in linking sleep and epilepsy. Biologically, *SPAG7* (sperm associated antigen 7) has been implicated in cellular stress, inflammatory responses and immune-related pathways [64]. Its robust expression across the hippocampus, frontal cortex (BA9), and cerebellar regions suggests a potential role in biological processes relevant to both sleep regulation and epilepsy. Its expression in the hippocampus and frontal cortex, regions involved in both sleep–wake regulation and epileptogenesis, suggests that *SPAG7* may contribute to biological pathways underlying the genetic overlap between sleep traits and epilepsy. Conversely, *VRK2* encodes a serine-threonine kinase that modulates MAPK signaling and neuronal proliferation and migration. In our TWAS analysis, *VRK2* expression was prioritized in the substantia nigra, a basal ganglia structure involved in REM sleep regulation and the gating of seizure propagation. And *VRK2* may influence pathways regulating neuronal excitability and synaptic function, thereby contributing to biological mechanisms shared between sleep disturbances and epilepsy [58]. Beyond *SPAG7* and *VRK2*, our cross-trait meta-analysis identified several other critical pleiotropic loci, including *CNIH2*, *YIF1A*, and *LINC00925*. Correlating these loci with our pathway enrichment results, which highlighted membrane trafficking and organelle biogenesis, provides a cohesive biological hypothesis. Specifically, *CNIH2* regulates AMPA receptor trafficking [59], while *YIF1A* maintains Golgi apparatus architecture [60]. These findings suggest that genetic dysregulation at these loci may influence intracellular trafficking and excitatory neurotransmission. These mechanisms may contribute to alterations in neuronal function that are relevant to both sleep regulation and seizure susceptibility. Moreover, these pathway enrichments suggest a critical neurobiological convergence linking circadian rhythm regulation directly to seizure susceptibility. Disruptions in the expression of key genes may alter the intrinsic circadian oscillations of neuronal networks, lowering the threshold for epileptiform activity during specific sleep stages [63,65,66]. To conceptualize these convergent pathways, we propose a schematic model illustrating the shared genetic architecture linking sleep architecture fragmentation with epileptogenesis (Graphical Abstract). To transition these statistical findings into testable biological models, future in vitro studies should utilize patient-derived induced pluripotent stem cells (iPSCs) differentiated into cortical neurons to assess AMPA receptor surface dynamics. Concurrently, in vivo knockout murine models could be employed to evaluate behavioral seizure susceptibility and electroencephalogram (EEG) sleep spindle abnormalities. Recent advances in computational biology and neuro-informatics have increasingly highlighted the value of integrating large-scale omics data to identify novel diagnostic and therapeutic biomarkers for epilepsy [67]. In this context, *LINC00925*, a long non-coding RNA (lncRNA), has been linked to cardiomyocyte ferroptosis [62], emerging as a potential genetic biomarker or mechanistic candidate. To evaluate translational viability, we queried PheWAS databases. While our initial profiling of these candidate targets showed no major adverse associations, we strictly emphasize that the absence of evidence in genetic databases is not evidence of clinical safety, and rigorous pharmacological validation remains essential.

Finally, our findings indicate a potential causal relationship between short sleep duration and epilepsy. Specifically, epilepsy appears to increase the risk of short sleep duration, whereas the reverse causal effect was not supported. The genetic and neurobiological convergence observed between short sleep duration and epilepsy may involve mechanisms such as cortical excitability, arousal system overactivity [68], sleep regulation [69], and GABAergic signaling [70]. Furthermore, the identification of shared candidate genes in specific brain regions, particularly the hippocampus, provides crucial mechanistic insights. Aberrant structural plasticity and neurogenesis in the hippocampus are fundamentally linked to epileptogenesis and represent critical targets for neurorepair and regenerative medicine [71]. Our findings suggest that the shared genetic liability may locally disrupt these neural circuits, thereby modifying both sleep architecture and seizure susceptibility. Although the estimated causal effect size (BETA = 0.0006) appears numerically small, it reflects the impact of lifelong genetic liability rather than an acute or large-magnitude clinical intervention. It is crucial to interpret these findings cautiously, as such small effect sizes are characteristic of highly polygenic traits. Crucially, the statistically significant MR findings support a potential causal effect of genetic liability to epilepsy on short sleep duration, validating this axis as a potential focus for future mechanistic research. Conversely, our results suggest that neither long sleep duration nor insomnia causally contributes to the development of epilepsy. The differential genetic associations observed for short versus long sleep duration likely reflect their distinct underlying neurobiological architectures. Short sleep duration is frequently driven by hyperarousal and elevated neuronal excitability, and pathophysiological states that may overlap with mechanisms involved in seizure generation. In contrast, long sleep duration is more commonly associated with systemic inflammation, metabolic dysregulation, and psychiatric comorbidities such as depression, which possess a largely divergent genetic basis from epileptogenesis.

Several limitations exist in this study. First, to mitigate population stratification, we used GWAS data restricted to individuals of European ancestry, leaving genetic relationships between sleep behaviors and epilepsy in other populations unexplored. Therefore, prioritizing replication in diverse, non-European cohorts is essential to establish the global generalizability of these findings. Second, the insomnia phenotype was derived from a single self-reported questionnaire item, introducing a potential risk of misclassification bias compared to clinically validated diagnoses. Future investigations should integrate objective measurements, such as polysomnography or actigraphy-based phenotyping, to refine these clinical sleep traits. Third, we assessed local genetic correlations among sleep duration, insomnia, and epilepsy across 1703 genomic regions using only the GWAS-pw method. The lack of significant local correlations may reflect distinct causal variants within the same loci, highlighting the need for validation with advanced tools such as SUPERNOVA [72]. Fourth, our TWAS identified shared genes only in whole blood and brain tissues. Given the roles of other tissues in disease, future research should explore additional tissue types. Finally, this study relies fundamentally on summary-level statistics and lacks adjustments for longitudinal data or environmental covariates. Given these constraints, our findings must be interpreted cautiously as hypothesis-generating statistical associations rather than clinically actionable targets. To address these limitations, future research should aim to integrate multi-omics layers, such as epigenomics and metabolomics, alongside longitudinal sleep monitoring. Future experimental studies and integrative analyses utilizing large-scale, diverse single-cell omics repositories [73] will be crucial to pinpoint the specific neural cell populations driving these associations and to fully unravel the genetic links between sleep behaviors and epilepsy. Additionally, establishing a structured roadmap for functional validation using in vitro neuronal cell models and in vivo animal epilepsy models remains essential to confirm the translational credibility of these shared genetic pathways.

## 5. Conclusions

In conclusion, our multi-omics analysis provides robust statistical evidence for a shared genetic architecture between sleep traits and epilepsy. By integrating cross-trait meta-analysis and TWAS frameworks, we identified significant genetic correlations and specific shared loci. Notably, *SPAG7* and *VRK2* emerged as pleiotropic candidate genes implicated in neuroimmune and synaptic regulation, warranting further functional investigation. Furthermore, bidirectional MR suggested a unidirectional causal liability from epilepsy to short sleep duration; however, the extremely small magnitude of this effect size (BETA = 0.0006) reflects a subtle polygenic influence rather than an acute clinical driver. It is imperative to interpret these findings in light of several critical limitations, including the strict restriction to European ancestry datasets, the reliance on crude self-reported sleep phenotypes, the use of summary-level statistics, and the absence of independent cohort replication. Consequently, while our study highlights shared genetic pathways, we emphasize that these statistical associations do not immediately translate to clinical applications. Rigorous in vitro and in vivo experimental validations are strictly required before any of these candidate genes can be considered for future therapeutic strategies.

## Data Availability

We used publicly available datasets in this study. Short sleep duration and long sleep duration GWAS datasets can be downloaded at the Sleep Disorder Knowledge Portal (accessed on 21 May 2025). Insomnia GWAS datasets can be downloaded at CTGlab (https://cncr.nl/ctg/, accessed on 21 May 2025). The epilepsy GWAS summary dataset was obtained from https://www.ebi.ac.uk/gwas/ (accession IDs: GCST90271611, accessed on 21 May 2025).

## Supplementary Materials

The following supporting information can be downloaded at: https://www.mdpi.com/article/10.3390/biology15110892/s1, Table S1: overview of sleep duration and migraine diseases included in this study; Table S2: genome-wide genetic correlations between sleep traits and epilepsy by using constrained and unconstrained LDSC; Table S3: partitioned genetic correlations between short sleep duration and epilepsy; Table S4: partitioned genetic correlations between long sleep duration and epilepsy; Table S5: partitioned genetic correlations between insomnia and epilepsy; Table S6: local genetic correlations between short sleep duration and epilepsy by using GWAS-pw analysis; Table S7: local genetic correlations between long sleep duration and epilepsy by using GWAS-pw analysis; Table S8: local genetic correlations between insomnia and epilepsy by using GWAS-pw analysis; Table S9: Independent pleiotropic genomic loci associated with the risk of short sleep duration and epilepsy (*P*_CPASSOC_ < 5 × 10^−8^, *P*_single-trait_ < 0.05); Table S10: Independent pleiotropic genomic loci associated with the risk of long sleep duration and epilepsy (*P*_CPASSOC_ < 5 × 10^−8^, *P*_single-trait_ < 0.05); Table S11: Independent pleiotropic genomic loci associated with the risk of insomnia and epilepsy (*P*_CPASSOC_ < 5 × 10^−8^, *P*_single-trait_ < 0.05); Table S12: Top 5 clusters with representative enriched terms based on annotated genes between short sleep duration and epilepsy by Metascape; Table S13: Top 6 clusters with representative enriched terms based on annotated genes between long sleep duration and epilepsy by Metascape; Table S14: Top 1 cluster with representative enriched terms based on annotated genes between insomnia and epilepsy by Metascape; Table S15: TWAS revealed the significant shared genes between sleep behavior and epilepsy traits; Table S16: Candidate therapeutic drugs for potential protein targets provided by DGIdb and DSigDB; Table S17. Sensitivity analyses for bidirectional MR between sleep traits and epilepsy.
